# *Eryngium caeruleum*: an update on ethnobotany, phytochemistry and biomedical applications

**DOI:** 10.1186/s13020-022-00672-x

**Published:** 2022-09-29

**Authors:** Dmitryi Alexeevich Konovalov, Edgardo Avendaño Cáceres, Ekaterina Aleksandrovna Shcherbakova, Jesús Herrera-Bravo, Deepak Chandran, Miquel Martorell, Muzaffar Hasan, Manoj Kumar, Saad Bakrim, Abdelhakim Bouyahya, William C. Cho, Javad Sharifi-Rad, Hafiz A. R. Suleria, Daniela Calina

**Affiliations:** 1 Pyatigorsk Medical-Pharmaceutical Institute, Branch of Volgograd State Medical University, Volgograd, Russia; 2 Departamento de Química e Ingeniería Química, Facultad de Ingeniería, Universidad Nacional Jorge Basadre Grohman, Av. Miraflores s/n, Tacna, 23001 Perú; 3 Departamento de Ciencias Básicas, Facultad de Ciencias, Universidad Santo Tomas, Santiago, Chile; 4 Center of Molecular Biology and Pharmacogenetics, Scientific and Technological Bioresource Nucleus, Universidad de La Frontera, 4811230 Temuco, Chile; 5 Department of Veterinary Sciences and Animal Husbandry, Amrita School of Agricultural Sciences, Amrita Vishwa Vidyapeetham University, Coimbatore, 642109 Tamil Nadu India; 6 Department of Nutrition and Dietetics, Faculty of Pharmacy, and Centre for Healthy Living, University of Concepción, 4070386 Concepción, Chile; 7 Universidad de Concepción, Unidad de Desarrollo Tecnológico, UDT, 4070386 Concepción, Chile; 8 Agro Produce Processing Division, ICAR - Central Institute of Agricultural Engineering, Bhopal, 462038 India; 9 Chemical and Biochemical Processing Division, ICAR - Central Institute for Research on Cotton Technology, Mumbai, 400019 India; 10 Geo-Bio-Environment Engineering and Innovation Laboratory, Molecular Engineering, Biotechnologies, and Innovation Team, Polydisciplinary Faculty of Taroudant, Ibn Zohr University, Agadir, Morocco; 11 Laboratory of Human Pathologies Biology, Department of Biology, Faculty of Sciences, Mohammed V University in Rabat, Rabat, Morocco; 12 Department of Clinical Oncology, Queen Elizabeth Hospital, Kowloon, Hong Kong; 13 Facultad de Medicina, Universidad del Azuay, Cuenca, Ecuador; 14 School of Agriculture and Food, Faculty of Veterinary and Agricultural Sciences, The University of Melbourne, Parkville, VIC 3010 Australia; 15 Department of Toxicology, University of Medicine and Pharmacy of Craiova, 200349 Craiova, Romania

**Keywords:** *Eryngium caeruleum*, Ethnobotany, Bioactive molecules, Pharmacological activities

## Abstract

**Background:**

A biennial or perennial plant of the Apiaceae family, *Eryngium caeruleum* M. Bieb. is traditionally used in medicine as an antitoxic, diuretic, digestive, anti-inflammatory and analgesic drug. This plant is widely distributed in temperate regions around the world. Young leaves of the plant are used in cooking as aromatic cooked vegetables in various local products in Iran.

**Purpose:**

The current review aimed to highlight complete and updated information about the *Eryngium caeruleum* species, regarding botanical, ethnopharmacological, phytochemical data, pharmacological mechanisms as well as some nutritional properties. All this scientific evidence supports the use of this species in complementary medicine, thus opening new therapeutic perspectives for the treatment of some diseases.

**Methods:**

The information provided in this updated review is collected from several scientific databases such as PubMed/Medline, ScienceDirect, Mendeley, Scopus, Web of Science and Google Scholar. Ethnopharmacology books and various professional websites were also researched.

**Results:**

The phytochemical composition of the aerial parts and roots of *E. caeruleum* is represented by the components of essential oil (EO), phenolic compounds, saponins, protein, amino acids, fiber, carbohydrates, and mineral elements. The antioxidant, antimicrobial, antidiabetic, antihypoxic, and anti-inflammatory properties of *E. caeruleum* have been confirmed by pharmacological experiments with extracts using in vitro and in vivo methods. The syrup *E. caeruleum* relieved dysmenorrhea as effectively as Ibuprofen in the blinded, randomized, placebo-controlled clinical study.

**Conclusion:**

Current evidence from experimental pharmacological studies has shown that the different bioactive compounds present in the species *E. caeruleum* have multiple beneficial effects on human health, being potentially active in the treatment of many diseases. Thus, the traditional uses of this species are supported based on evidence. In future, translational and human clinical studies are necessary to establish effective therapeutic doses in humans.

## Introduction

The genus *Eryngium* L. (Apiaceae) has approximately 250 species, being the most species-rich genus of the Apiaceae, and is distributed worldwide in temperate regions. The *Eryngium* spp. grow in North Africa, Australia, North and South America, and Eurasia [55]. Two centres of diversity are recognized for the genus. The first one is central-east South America and central-west Mexico whereas the second is the western Mediterranean and south-west Asia [113]. Approximately two-thirds of *Eryngium* spp. are located in Central, South and North America [13].

The species were grouped by Wolff [117] into 34 sections and numerous subsections. *Eryngium caeruleum* M.Bieb. (syn. *Eryngium caucasicum* Trautv. and *Eryngium pskemense* Pavlov) belongs to Plana section [117]. Subgeneric classification based on the morphology of the genus was introduced by Wörz [119], which recognizes five subgenera. The study of Calviño et al. [12, 13], based on phylogenetic analyses of DNA sequences corroborated some of the hypotheses of relationships and biogeography previously formulated [12, 13]. *E. caeruleum* is very close to the Western Mediterranean *Eryngium dichotomum* Desf. from South Italy, Southern Spain, and Northwestern Africa [15, 53, 54].

**Fig. 1:**
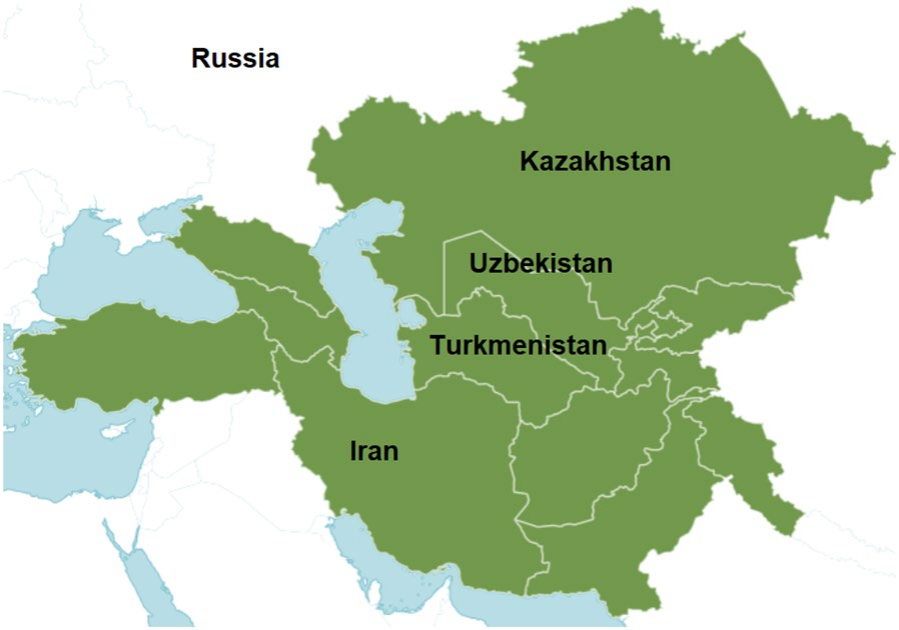
Distribution area of *Eryngium caeruleum* M. Bieb [74]

*Eryngium caeruleum* is an Oriental-Caucasian-Turcestanian floral component [58] that is widespread from North-Eastern Anatolia, Caucasia and Southern Russia across Central, Northern, Eastern Iran and Middle Asia (Southern Turkmenistan, Tyan Shan, Pamir-Alaj) to Afghanistan, Pakistan, and Western Himalaya [15, 54, 70, 71, 118]. The plant is indigenous to Kazakhstan, Afghanistan, Iran, North Caucasus, Kirgizstan, Tadzhikistan, Pakistan, Transcaucasus, Turkey, Uzbekistan, Turkmenistan, West Himalaya (Fig. 1) [74].

Several bioactive molecules, principally essential oils, terpenoids and phenolics, have been identified and purified from different parts of *E. caeruleum*, such as flavonoids and saponins [21, 41, 62]. Antioxidant, antidiabetic, antihypoxic, anticonvulsant, free radical scavenging, hepato-renoprotective, reproductive protection, and anti-inflammatory effects of *E. caeruleum* were reported [18, 23, 45, 46, 52, 59, 66, 66, 80]. For these reasons, it is important to note that understanding the bioactive constituents of plants that play an essential role in human healthcare, as well as scientific proof of their traditional use, should be made available to support traditional medicine and herbal treatments. The geographical pattern and botanical description of *E. caeruleum*, are discussed, as well as phytochemicals constituents, ethnopharmacology, biological activities and clinical studies are also more detailed.

In summary, this review summarizes the findings of previous *E. caeruleum* research and identifies information gaps that must be addressed to fully comprehend the mode of action of extracts used in traditional medical practice. It also suggests that a thorough investigation of the relationship between the pharmacological impact and the existence of the bioactive chemicals responsible for the action is required. The review synthesizes phytochemical analyses and biological studies that may be useful for researchers to conduct additional studies to supplement existing information and promote the discovery of new uses for these fascinating species.

## Review methodology

Scientific databases and repositories, such as PubMed/Medline, ScienceDirect, Mendeley, Scopus, Web of Science and Google Scholar were used to search for information. The research was carried out using the next MeSH terms: Eryngium/classification, Eryngium /chemistry, Eryngium/Flavonoids, Plant Extracts/pharmacology, Plants, Medicinal, Anti-Infective Agents/pharmacology, Plant Oils/pharmacology. We obtained about 993 references according to these terms. References relating to *E. caeruleum* consist of 28 English and 15 Russian sources.

Inclusion criteria: publications which have focused on botanical description, geographical repartition, traditional uses, chemistry and pharmacological activities of *E. caeruleum*. These data have been arranged in tables and presented in this study according to each field.

Exclusion criteria: articles written in other languages than English, papers without pharmacological mechanisms included, meta-analyses, case studies or works that included pharmacological experiments associated with homoeopath products.

The most important data were summarized in tables and figures. The taxonomy of plants has been validated using the PlantList and chemical formulas according to PubChem [38, 72].

## Botany

*Eryngium caeruleum* is a biennial or plurennial hapaxanthic plant turning glaucous-amethyst into a flower. Synflorescence (40–80 cm) has a dichotomous branch structure with spreading horizontal branches. The plant has basal leaves herbaceous, and long petiolate; the outer leaves decay early. The median cauline leaves are stalkless and profoundly palmate in spiny pinnatifid lobes [54]. In many tropical areas of the world, this genus has been extensively employed as an eatable plant and grown as an economic crop [27, 66, 69]. Cultivation was begun in some domestic gardens in Shirkala (Iran) and the undivided outer basal leaves are collected continuously in spring and summer to prevent flowering [54]. In the northern area of Iran, *E. caeruleum* is among the most prominent garden vegetables [27, 66] because it is easily grown in sandy, well-drained, dry soils, and under the full sun [25, 54, 64, 69]. Eliseeva et al. [24] studied the possibility of cultivating the *E. caeruleum* on the territory of the Caucasian Mineral Waters (North Caucasus, Russia). Seeds were sown at different times (in March and November), and the distance between the rows is 40–60 cm. Seeds germinated without a dormant period. Shoots appeared after 2–4 weeks, depending on climatic conditions. Seed germination ranged from 39 to 55% [24]. In the life cycle of *E. caeruleum*, the investigating authors identified several stages of development: germinant, juvenile, immature, adult vegetative and generative plants. The germinant had a height of up to 1 cm. The cotyledonous leaves are oval, up to 1.3 cm long, and up to 0.7 cm wide. The root has a length of up to 2 cm. At this stage, plants are 7–10 days. Juvenile plants have a height of 1.3–1.5 cm; the number of leaves is 2–3. Immature plants are characterized by the appearance of real leaves in the amount of 12–45. Leaves are parallel to the surface of the soil. Adult vegetative plants are fully formed on the 50–57 day of vegetation. They have a rosette of leaves with a diameter of 50–60 cm. The main root is 20–25 cm long, and 1.5–1.8 cm thick. The generative period of development is observed in the second year of vegetation. One to five peduncles are formed [24, 103]. The flowering period of plants was observed from May to July (40–50 days), plants reach up to 100–120 cm in height, and stems give an increase of 15–20 cm per week. *E. caeruleum* forms fruits in July–September. Fruits and seeds usually ripen in late August-early September [104]. Crop capacity (yield of raw materials from 1 m^2^) is on average: 0.72 kg for roots, overhead mass—1.2 kg, seeds—about 40 g [102]. The weight of the fresh aerial mass of one plant is on average 326.5 g. The roots for the second year are 38–45 cm long, 2.1–4.5 cm thick, an average weight of 117.6 g, seeds from one plant can produce up to 24.8 g [103].

## Traditional and ethnomedicinal importance

The fresh leaves of *E. caeruleum* are used to flavor cooked vegetables in various local products of Iran and are also often used to flavor chicken and fish. Also, cooked leaves are mixed with yoghurt or used in soups [7, 54]. In Persian medicine, various therapeutic effects, and pharmacological actions of *Eryngium* had mentioned, including antitoxic, diuretic, emmenagogue, aphrodisiac, galactagogue, digestive, anti-flatulent, anti-inflammatory and analgesic properties. This plant was used for pulmonary diseases such as asthma and bronchitis, halitosis, snakebite and insect bites, cramps and influenza, early stages of lymphatic filariasis, hypochondriac, and epigastric pain [88]. The roots of the plant have a diuretic effect, a beneficial effect on vesical and kidney stones, as well as on peripheral oedemas. In traditional Iranian medicine, *E. caeruleum* increases lactation and sweating, as carminative, an aromatic agent and a fragrant. It decreases oedema and inflammation, promotes digestion, and removes phlegm from the digestive system [7, 8, 29]. The plant is currently of great scientific interest due to its historic use. *E. caeruleum* is also used to cure a variety of diseases in Islamic medicine; the roots, in particular, are used to treat inflammations, edema, urinary infections, and sinusitis [55]. In China, this plant is also used to treat cough, malaria, and animal poisoning [3]. The roots are used as an analgesic for rheumatoid arthritis patients [33].

## Phytochemistry

### Essential oils

Essential oils are concentrated hydrophobic liquids containing volatile chemical compounds from plants [93, 97, 99, 101]. Studies by various authors have established that the content of essential oils (Eos) in the aerial part of *E. caeruleum* is from 0.053 to 1.1% v/dry weight and increases during the growth and development of plants, reaching maximum values in the flowering phase. In the roots—up to 1.1 v/dry weight. The composition of EOs of dried samples of the aerial parts and roots of *E. caeruleum* growing in Iran according to Mohamadipour et al. [61] and Hamedi et al. [35] very close. As described, trans-pinocarvyl acetate and short-chain fatty acid esters are among the main components [35, 61]. It is noteworthy that the content of the polyacetylene compound Z-falcarinol in the composition of EOs derived from the roots and aerial parts of the plant is approximately the same (5.5% v/dry weight and 5.6% v/dry weight). Studies by Morteza-Semnani et al. [62], Assadian et al. [5] and Saeedi and Morteza-Semnani [81] found that limonene (52.1–60.5%), α-pinene (5.5–6.5%) and δ-2-carene (5.3–13.0%) accumulate as the main ingredients in the EO of the aerial parts of plants from Iran.

A study by Hashemabadi and Kaviani [36] showed that the composition of EOs from the leaves and stems of coastal and hill slope plants varies in different phases of their development. Unlike previous data, the main components of the EOs were sesquiterpenes (see Table 1). According to the authors, this study remarks that type and concentration of phytochemicals can be changed, based on the vegetation phase and location of *E. caeruleum.*

**Table 1:** Essential oil analysis of *Eryngium caeruleum* M.Bieb

Origin of materials (country, province, locality)/part used/phase of development	Method of extraction (extraction time)/yield (%)	Main components	References
Iran/aerial parts	Hydrodistillation	Limonene (52.1%), β-sesquiphellandrene (8.1%), α-pinene (5.5%) and δ-2-carene (5.3%)	[62]
Iran/aerial parts/flowering stage	Hydrodistillation (3 h)/0.3 air-dried weight	Limonene (60.5%), δ-3-carene (13.0%), α-pinene (5.6%)	[5]
Iran/dried aerial parts	Hydrodistillation (5 h)/0.65 w/w	Limonene (56.7%), β-sesquiphellandrene (8.9%), α-pinene (6.5%), δ-2-carene (5.9%)	[81]
Iran/dry leaves/vegetative phase	Hydrodistillation (4 h)	Coastal plants: 5-methyl-2-pyrimidone (61.69%), β-sesquiphellandrene (14.19%), 2,4-bis (1,1-dimethyl)-phenol (13.65%)	[36]
Iran/dry leaves/vegetative phase		Hill slope plants: 5-methyl-2-pyrimidone (41.74%), β-sesquiphellandrene (18.29%), β-bisabolene (12.30%)	
Iran/dry stems/vegetative phase		Coastal plants: l-limonene (30.34%), β-sesquiphellandrene (28.53%), β-bisabolene (15.31%)	
Iran/dry stems/vegetative phase		Hill slope plants: β-sesquiphellandrene (26.54%), 5-methyl-2-pyrimidone (23.89%), limonene (11.57%)	
Iran/leaves/first vegetative stages	Hydrodistillation (4 h)	Coastal plants: β-sesquiphellandrene (44.21%), limonene (18.39%) and β-bisabolene (6.08%)	[36, 37]
		Hill slope plants: 5-methyl-2-pyrimidone (53.83%), β-sesquiphellandrene (11.26%) and β-bisabolene (7.43%)	
Iran/leaves/second vegetative phase		Coastal plants: β-sesquiphellandrene (27.32%), limonene (14.32%) and 5-methyl-2-pyrimidone (14.15%)	
		Hill slope plant: 4(5)-acetyl-1H-imidazole (50.14%), β-sesquiphellandrene (15.51%) and 4-(1,5-dimethylhex-4-enyl) cyclohex-2-enone (11.05%)	
Iran/stems/generative phase		Coastal plants: hexadecahydrocyclobuta [1,2,3,4] dicyclooctene (45.46%), β-sesquiphellandrene (20.5%) and widdrene (19.06%)	
		Hill slope plant: piperiton (69.81%), 4-(1,5-dimethylhex-4-enyl) cyclohex-2-enone (18.38%) and β-sesquiphellandrene (4.54%)	
Iran/dry leaves/pre-flowering stage	Hydrodistillation (3 h)	Cyclobuta, dicyclooctene, hexadecahydro (47.03%), n-hexadecanoic acid (11.16%), linoleic acid (5.41%), limonene (4.23%), cis-α-bisabolene (2.14%)	[18]
Iran/fresh flowers/early reproductive phase	Hydrodistillation/0.32	Littoral location: allo-aromadendrene (66.3%), trans-calamenene (11%), dehydro abietal (6.7%), α-calacorene (6.1%)	[1]
Iran/fresh flowers/early reproductive phase	Hydrodistillation/0.38	Unlittoral location: allo-aromadendrene (61.2%), trans-calamenene (13.4%), dehydro abietal (10.9%)	
Iran/fresh flowers/early reproductive phase	Hydro-steam distillation/0.176	Littoral location: allo-aromadendrene (71.6%), trans-calamenene (12.8%), α-calacorene (4.5%)	
Iran/fresh flowers/early reproductive phase	Hydro-steam distillation/0.21	Unlittoral location: allo-aromadendrene (69.1%), trans-calamenene (15.9%), dehydro abietal (4.5%)	
Iran/fresh flowers/early reproductive phase	Steam distillation/0.06	Littoral location: allo-aromadendrene (48.7%), trans-calamenene (11.1%), α –eudesmol (4.4%), dehydro abietal (4.0%)	
Iran/fresh flowers/early reproductive phase	Steam distillation/0.09	Unlittoral location: allo-aromadendrene (56.7%), trans-calamenene (18.2%), dehydro abietal (8.5%)	
Iran/fresh leaves/early reproductive phase	Hydrodistillation/0.13	Littoral location: (E, E)-farnesol (24.3%), elemicin (12.2%), n-nonanyl acetate (9.1%), butyl acetate (6.4%), allo-aromadendrene (6.0%)	
Iran/fresh leaves/early reproductive phase	Hydrodistillation/0.19	Unlittoral location: allo-aromadenderene (25.2%), α-calacorene (23.1%), (E,E)-farnesol (17.5%), dihydro tagetone (11.0%)	
Iran/dry leaves/early reproductive phase	Hydrodistillation/0.17	Littoral location: alloaromadendrene (24%), dihydro tagetone (19.8%), (E,E)-farnesol (13.8%), elemicin (12.3%)	
Iran/dry leaves/early reproductive phase	Hydrodistillation/0.32	Unlittoral location: allo-aromadenderene (33.2%), α-calacorene (14.4%), (E,E)-farnesol (16.7%), dihydro tagetone (8.3%)	
Iran/fresh leaves/early reproductive phase	Hydro-steam distillation/0.1	Littoral location: allo-aromadenderene (30.6%), (E,E)- farnesol (28.3%), dihydro tagetone (12%)	
Iran/fresh leaves/early reproductive phase	Hydro-steam distillation/0.16	Unlittoral location: allo-aromadenderene (30.3%), (E,E)-farnesol (29.1%), α-calacorene (11.5%)	
Iran/dry leaves/early reproductive phase	Hydro-steam distillation/0.1	Littoral location: allo-aromadendrene (22.6%), dihydro tagetone (12.5%), (E,E)-farnesol (16.0%), elemicin (14.1%), methyl octadecanoate (10.9%)	
Iran/dry leaves/early reproductive phase	Hydro-steam distillation/0.16	Unlittoral location: allo-aromadenderene (30.8%), dihydro tagetone (17.9%), (E,E)-farnesol (13.5%), α-calacorene (11.6%)	
Iran/fresh leaves/early reproductive phase	Steam distillation/0.1	Littoral location: 1-phenyl pentan-3-one (23.8%), trans-chrysanthenyl acetate (11.3%), dihydro tagetone (8.7%), α-ylangene (8.5%)	
Iran/fresh leaves/early reproductive phase	Steam distillation/0.14	Unlittoral location: allo-aromadenderene (13.0%), n-dodecanol (9.0%), (E,E)-farnesol (12.1%), α-calacorene (7.7%), methyl octadecanoate (7%)	
Iran/dry leaves/early reproductive phase	Steam distillation/0.053	Littoral location: allo-aromadenderene (26.4%), (E,E)-farnesol (18%), elemicin (10.5%), methyl octadecanoate (8.3%)	
Iran/dry leaves/early reproductive phase	Steam distillation/0.087	Unlittoral location: alloaromadenderene (32.3%), α-calacorene (15.5%), (E,E)-farnesol (11.2%)	
Iran/stems/early reproductive phase	Hydrodistillation/0.18	Littoral location: allo-aromadenderene (56.4%), trans-calamenene (12%), dihydro tagetone (9.4%), dehydro abietal (9.6%)	
Iran/stems/early reproductive phase	Hydrodistillation/0.2	Unlittoral location: allo-aromadenderene (36%), trans-calamenene (12.6%), dihydro tagetone (6.4%), dehydro abietal (31.5%)	
Iran/stems/early reproductive phase	Hydro-steam distillation/0.11	Littoral location: allo-aromadenderene (55.5%), methyl octadecanoate (20.2%), trans-calamenene (8.3%), α- calacorene (5.8%)	
Iran/stems/early reproductive phase	Hydro-steam distillation/0.14	Unlittoral location: alloaromadenderene (47.4%), dehydro abietal (19.5%), trans-calamenene (14.7%), dihydro tagetone (6.9%)	
Iran/stems/early reproductive phase	Steam distillation/0.09	Littoral location: alloaromadenderene (67.4%), trans-calamenene (11%), dehydro abietal (6.3%), α- calacorene (6.0%)	
Iran/stems/early reproductive phase	Steam distillation/0.1	Unlittoral location: allo-aromadenderene (54.9%), trans-calamenene (16.2%), dehydro abietal (11.7%), elemicin (3.9%)	
Iran/roots/early reproductive phase	Hydrodistillation/0.50	Littoral location: n-octadecanol (91%), n-nonadecane (1.2%), 1-butyl acetate (1.2%)	
Iran/roots/early reproductive phase	Hydrodistillation/0.44	Unlittoral location: n-octadecanol (95.6%), abietatriene (1.7%)	
Iran/roots/early reproductive phase	Hydro-steam distillation/0.33	Littoral location: n-octadecanol (73.8%), 1-butyl acetate (4%) and trans-chrysanthenyl acetate (3.6%)	
Iran/roots/early reproductive phase	Hydro-steam distillation/0.28	Unlittoral location: n-octadecanol (69.1%), α-terpinene (6.9%) and (E,E)-farnesol (5.2%)	
Iran/roots/early reproductive phase	Steam distillation/0.16	Littoral location: n-octadecanol (74.6%), dihydro tagetone (4.9%)	
Iran/roots/early reproductive phase	Steam distillation/0.15	Unlittoral location: n-octadecanol (43.5%), α-eudesmol (18.4%) and methyl octadecanoate (4.8%)	
Iran/fresh leaves/early reproductive phase	Hydrodistillation (3 h)/0.07	Limonene (26.71%), cyclobuta[1,2:3,4]dicyclooctene, hexadecahydro (24.19%), β-sesquiphellandrene (15.25%), δ-3-carene (6.79%), trans-longipinocarveo (5.28%), n-hexadecanoic acid (2.69%), Z-α-bisabolene (2.57%), myrcene (1.95%), α-pinene (1,87%), β-bisabolene (1.84%), n-octanal (1.53%), E-β-ionone (1.23%), vervenene (0.84%), widdrol (0.83%), α-cis-bergamotene (0.72%), n-octanol (0.54%), trans-carveol (0.53%), α-acoradiene (0.46%), linalool (0.42%), n-heptanol (0.42%), citronellol (0.38%), p-cymene (0.37%), carvacrol (0.35%), n-octadecane (0.28%), p-mentha-2,4(8)-diene (0.27%), thymol (0.23%), β-elemene (0.21%), cis-p-mentha-2,8-dien-1-ol (0.18%), myristicin (0.17%), benzene acetaldehyde (0.16%), heptanal (0.15%), Z-4-decenal (0.13%)	[6]
Iran/air-dried aerial parts/flowering phase	Hydrodistillation (3 h)/1.1 v/dry weight	Trans-pinocarvyl acetate (15.6%), caryophyllene oxide (13.2%), n-hexyl isobutyrate (11.9%), hexyl isovalerate (9.1%),	[61]
Iran/dry roots	Hydrodistillation (3 h)/1.1 v/dry weight	Hexyl isovalerate (11.0%), hexyl valerate (10.1%), hexyl isobutyrate (7.3%), octyl octanoate (7.0%), octyl isovalerate (6.8%), trans-pinocarvyl acetate (6.6%)	[35]

**Fig. 2:**
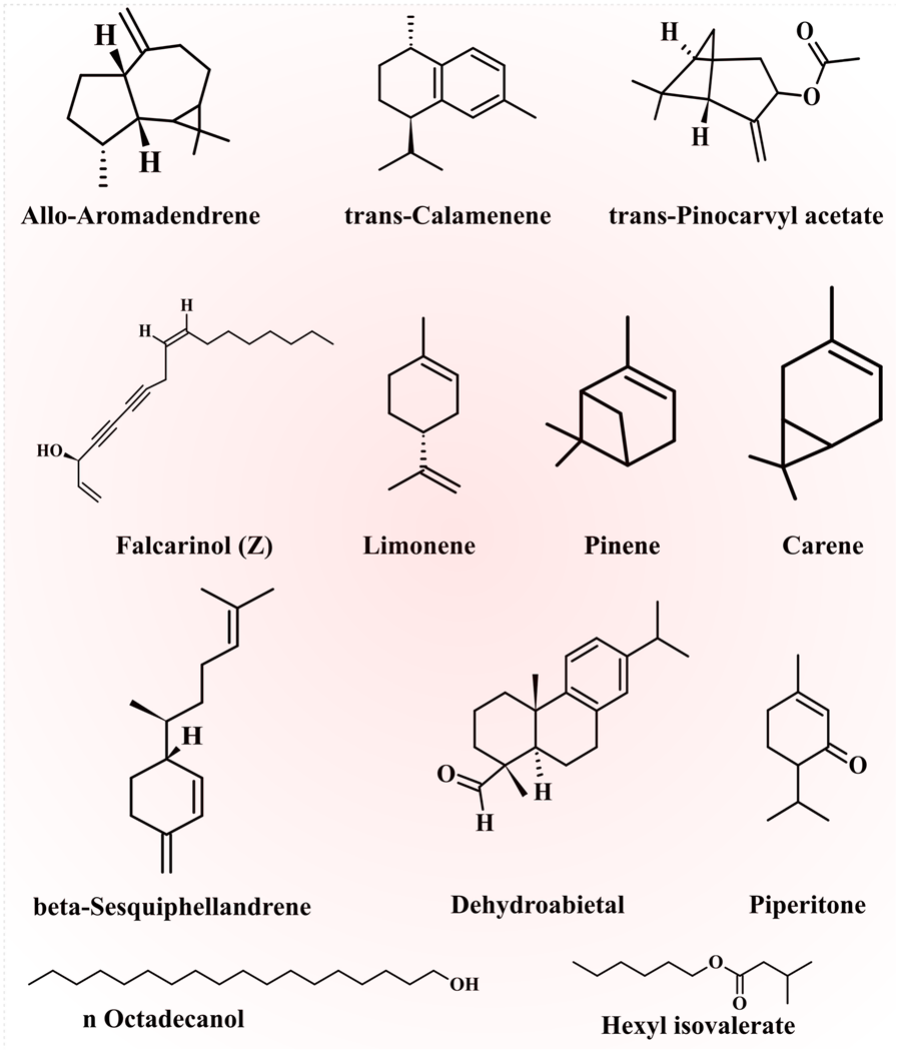
Chemical structure of essential oil constituents of *Eryngium caeruleum* M. Bieb

Dehghanzadeh et al. [18] isolated and identified EOs compositions from the aerial part of *E. caeruleum* at the pre-flowering stage and reported that among the fifty-six components cyclobuta[1,2,3,4]dicyclooctene, limonene (4.23%), n-hexadecanoic acid (11.16%), hexadecahydro (47.03%), linoleic (5.41%), and cis-α-bisabolene (2.14%) were the main components. Similarly, Mirahmadi et al. [59] also obtained EO from the aerial part of *E. caeruleum* and reported that twenty-three components contributed 96.7% of EO components and cyclobuta [1,2:3,4] dicyclooctene hexadecahydro (22.24%), limonene (25.42%), and δ-2-carene (16.19%) were the dominated components. Figure 2 shows the proven biological activities of the *E. caeruleum* extracts.

### Phenolic compounds

Phenols are a family of organic compounds (alcohols) that are characterized by the fact that they have an aromatic ring in which one or more of the hydrogen atoms associated with the carbon atoms of the ring are replaced by one or more hydroxyl groups (–OH) [56, 73, 92]. Phenols and their derivatives are normally present in nature [10, 47, 98, 100]. The main phenol compound from E. caeruleum is represented by gallic acid. Nabavi et al. [65] determined total phenolic compound contents in a crude solid methanol extract from *E. caeruleum* leaves using the Folin–Ciocalteau reagent. The total phenolic contents of *E. caeruleum* leaves was 62.3 mg gallic acid equivalent (GAE)/g of extract powder. The total flavonoid contents of *E. caeruleum* leaves was 25.3 mg quercetin equivalent (QE)/g of extract powder.In another study, total phenolic contents in methanol extracts of *E. caeruleum* leaves and inflorescences at the flowering stage were 37.6 and 63.1 mg GAE/g of extract powder, respectively [21]. In leaves and inflorescences were 60.0 and 18.3 mg QE/g of extract powder, respectively. The aqueous extract of *E. caeruleum* leaves reported 80.2 mg GAE/g and the total flavonoid of this extract powder was34.92 mg/g [22].

Many researchers correlated the antioxidant activity of *E. caeruleum* extracts with the content of phenolic compounds. Therefore Nabavi et al. [66] using the previously described methods determined the phenolic composition of a flavonoid-rich fraction. They found that total phenolic contents of hexane, ethyl acetate and aqueous fractions of *E. caeruleum* were 29.06, 140.57, 214.18 mg GAE/g of extract, respectively. The total flavonoid contents were 97.37, 31.51, and 75.36 mg QE/g of extract powder, respectively. The aqueous fraction exhibited higher levels of total flavonoids and phenol compared to the other fractions. Ebrahimzadeh et al. [21] performed a comparative study of phenolic content in methanol extracts from *E. caeruleum* inflorescences obtained by percolation, Soxhlet extractor, and ultrasonic method. Total phenolic contents ranged from 58.8 to 105.5 mg GAE/g of extract. Total phenolic contents appeared in the order of Soxhlet extract > percolation method > ultrasonic extract, respectively. The total flavonoid content was between 11.9 and 18.7 mg QE/g of extract being Soxhlet method the extraction method with higher flavonoid contents [21].In the same year, Dehghan et al. [16] published an article in which the authors cite data from their research of *E. caeruleum* aerial parts. The authors sequentially extracted the dried aerial part with hexane, ethyl acetate and methanol. The content of the sum of phenolic substances as mg GAE/g dry weight of *E. caeruleum* aerial parts is presented in Table 2. The highest content of phenolic compounds was found in methanol extract. Phytochemical investigation of methanol extract from aerial parts of *E. caeruleum* revealed the identification of two novel flavone glycosides. [79].

**Table 2:** The total phenolics and flavonoid contents of *Eryngium caeruleum* M.Bieb

Country of origin/part used/phase of development	Method of extraction/extractant	Total phenolic contents (gallic acid mg/g of extract powder)	Total flavonoid contents (quercetin equivalent mg/g of extract powder)	Refs.
Iran/leaves	Percolation/methanol	62.3 ± 0.2	25. 3 ± 0.2	[65]
Iran/leaves	Percolation/methanol	37.6 ± 1.5	63.1 ± 1.44	[21]
Iran/inflorescences	Percolation/methanol	60.0 ± 2.8	18.3 ± 0.9	
Iran/leaves	Percolation/water	80.2 ± 3.6	34.9 ± 1.1	[22]
Iran/leaves	Percolation/acetone, n-hexane	29.1 ± 1.8	97.4 ± 4.9	[66]
	Percolation/acetone, ethyl acetate	140.6 ± 6.5	31.5 ± 1.2	
	Percolation/acetone, water	214.2 ± 11.5	75.4 ± 3.6	
Iran/inflorescences	Ultrasonic/methanol	58.8 ± 1.5	18.2 ± 0.7	[21]
	Percolation/methanol	60.1 ± 2.3	11.9 ± 0.5	
	Soxhlet/methanol	105.5 ± 2.8	18.7 ± 0.9	
Iran/aerial parts	Percolation/n-hexane	7.1 ± 0.6		[16]
	Percolation/ethyl acetate	14.4 ± 3.3		
	Percolation/methanol	86.2 ± 5.0		

### Saponins

The main saponins of Eryngium species are represented by barrigenol, barringtogenol, cameliagenin, erynginol A, erynginol B, betulinic acid and steganogenin [26]. One of the goals of the study by Habibi et al. [34] was to determine the content of the total of saponins content in the aerial part of *E. caeruleum*. The raw material was collected mainly from the village of Kootena, on the outskirts of the city of Gaemshahr, Iran. According to the results of the research, the saponins content in the extract has been 5.4%.

## Nutritional properties

In the world of medicinal plants there are many nutritious plants [91, 109, 110]. The main objective is to provide the body with the necessary nutrients and to diversify their range [50, 57, 73, 77]. Medicinal plants have a high content of bioactive compounds, phytochemical molecules, vitamins, minerals, polyphenols, carotenoids, and can be compared very easily with the nutritional elements of fruits and vegetables [83, 90, 111, 114]. Along with these, herbs and natural bioactive compounds can optimize nutrition and provide optimal health [85, 96, 106].

In the study by Ebrahimzadeh et al. [22] the elemental composition of the leaves of *E. caeruleum* was investigated by atomic absorption spectroscopy, and the levels of Fe, Zn, Ni, Cu, Mn, and Cr were established [22]. The concentration of elements analyzed in the ash of *E. caeruleum* leaves (μg/g) decreased in the order: Fe (17.18) > Zn (0.83) > Mn (0.50) > Cr (0.41) > Cu (0.08). Of the four elements studied by Sepanlou et al. [89], Fe prevailed in the mineral composition of the leaves and roots of *E. caeruleum*. Autumn leaves showed the greatest amounts of Mn, Cu and, Zn while the roots contained more Fe [89]. Ash, protein, moisture, carbohydrate, fiber, and fat contents of the samples were determined from autumn leaves, spring leaves, and roots of *E. caeruleum* gathered in Gilan province, Iran [89]. The content of protein, fiber, carbohydrates, ash and moisture in various parts of *E. caeruleum* varied significantly. Autumn leaves compared with spring leaves showed the highest amount (% dry weight) of fiber (22.6 against 16.7), protein (17.9 against 16.9) and moisture contents (8.6 against 7.5). Spring leaves contained more carbohydrates (49.8 against 39.6) and fats (1.6 against 1.5). While roots – the highest levels of ash (10.1) and carbohydrates (55.9) in % of dry weight. Nine non-essential and eight essential amino acids were found in spring and autumn leaves and dried roots of *E. caeruleum*. The most dominant essential amino acid in all parts of *E. caeruleum* appeared to be threonine [89].

## Pharmacology: potential mechanisms of action

As evidenced in several preclinical studies in vitro, in vivo, *E. caeruleum* extracts or isolates attributed to different parts of this plant were revealed to exhibit a significant range of biological and pharmacological properties including antioxidant [22, 65, 66], antimicrobial [18, 46, 80], anti-diabetic anti-inflammatory, anticonvulsant [23], antihypoxic [52], and hepatic and renoprotective effects as well as other activities.

**Fig. 3:**
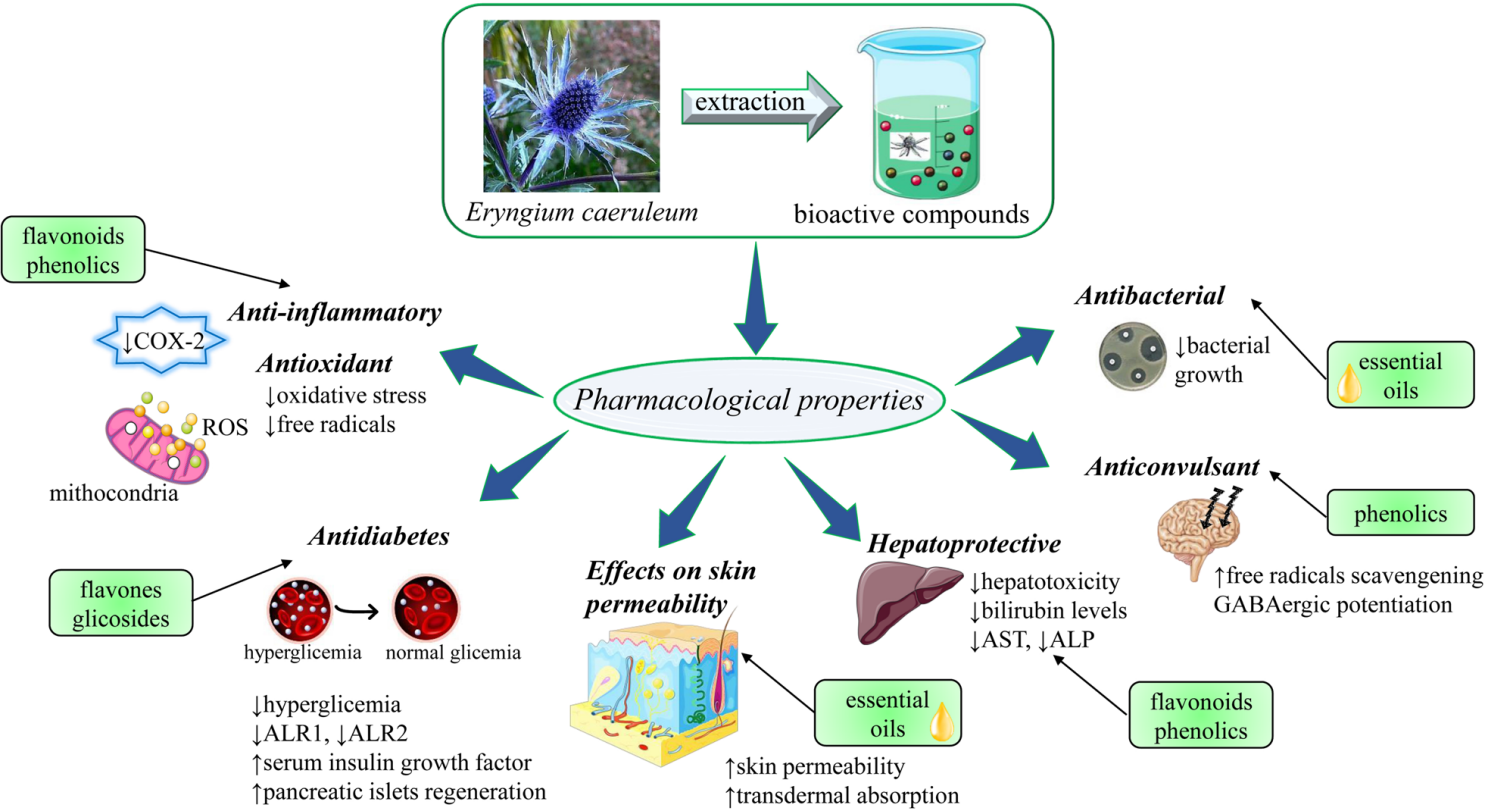
Summarized scheme with the most important activities and biomedical applications of *E. caeruleum* extracts and correlation with its bioactive compounds. ↓, decrease, aldose reductase (ALR), ↑, increase, *AST* aspartate aminotransferase, *ALP* alkaline phosphatase, *ROS* reactive oxygen species, *COX-2* cyclooxygenase-2

Figure 3 shows the proven biological activities of the *E. caeruleum* extracts.

### Antioxidant

Oxidative stress is one of the leading causes of cellular aging and the onset of chronic diseases [56, 67, 87]. Oxidative stress occurs as a result of an imbalance between free radicals and antioxidants [19, 45, 60, 121]. Natural antioxidants are substances that neutralize or eliminate free radicals by donating an electron [95, 112]. The neutralizing effect of antioxidants helps protect the body from oxidative stress [46, 60, 83, 99]. The antioxidant activity of *E. caeruleum* leaves (collected from Mazandaran forest) was investigated by Nabavi et al. [65] using a variety of in vitro test systems. For the methanol extract *E. caeruleum* leaves, the half-maximal inhibitory concentration (IC_50_) for DPPH (1,1-diphenyl-2-picryl hydrazyl radical) radical-scavenging activity was 0.27 mg/mL. The reducing power of methanol extract was equivalent to that of vitamin C (positive control, p > 0.05). The extract exhibited low Fe2+ chelation and nitric oxide-scavenging properties. The peroxidation inhibition by *E. caeruleum* extracts exhibited values from 93 (at 24 h) to 97% (at 72 h), comparable with vitamin C. The extract did not show good scavenging activity of hydrogen peroxide [65]. In another study, the antioxidant activities of inflorescence and leaf methanol extracts of *E. caeruleum* (collected from Khazar Abad area) were analyzed [21]. IC_50_ for DPPH assay was 0.39 mg/mL for inflorescences and 0.15 for leaves. The leaf extract demonstrated higher potency compared to vitamin C (p < 0.05). In nitric oxide-scavenging activity, methanol extracts showed weak activities. The leaf extract exhibited improved Fe2 + chelating capacity (IC_50_ = 0.25 mg/mL) that was similar to that of EDTA (ethylenediaminetetraacetic acid; IC_50_ = 18 μg/mL). Inflorescence extract was found to have very low potency. For hydrogen peroxide assay, IC_50_ was 177.2 and 25.5 mg/mL for inflorescence and leaves, respectively. Continuing their research Ebrahimzadeh et al. [22] examined the antioxidant activity of aqueous extract obtained from the leaves of *E. caeruleum* (collected from Panbeh Chooleh, Sari, Iran). IC_50_ for DPPH radical-scavenging activity of butylated hydroxyanisole (BHA) was 53.96, quercetin—5.28, and vitamin C—5.05 µg/mL. They found that IC_50_ for DPPH radical-scavenging activity of aqueous extract *E. caeruleum* was 7.99 mg/mL. The extract's reducing power also improved as its concentration increased but was found to be less than that of vitamin C. The activity of quercetin was more significant compared to the aqueous extract of *E. caeruleum* as evidenced by nitric oxide-scavenging activity analysis.

The leaf extract of *E. caeruleum* demonstrated an effective chelating ability, however, it could not be compared with that of EDTA (p < 0.01). For hydrogen peroxide scavenging activity, IC_50_ for quercetin and vitamin C were 52 and 21.4 mg/mL, respectively, which were higher than the extract of *E. caeruleum* (IC_50_ = 0.80 mg/mL). The investigated extract did not exhibit any activity in peroxidation inhibition in ferric thiocyanate assay in comparison with BHA and vitamin C used as controls [22]. The antioxidant effect of the flavonoid-rich part of this plant was subsequently examined [66]. Aqueous, ethyl acetate and n-hexane fractions showed an IC _50_ values for DPPH scavenging activity in the range of 391.2, 706.6 and 779.7 μg/mL, respectively. IC_50_ for nitric oxide radical-scavenging activity was in the order: aqueous (133.5 μg/mL) > ethyl acetate (350.1 μg/mL) > and hexane (639.9 μg/mL) fractions, respectively. The extracts showed low hydrogen peroxide scavenging activity but excellent antioxidant effect against hemoglobin-induced peroxidation of linoleic acid. The fractions have delayed the initiation of hemolysis induced by cumene hydroperoxide. The study of ethanol and methanol extracts from the aerial parts, inflorescences and leaves of *E. caeruleum*, as well as their hexane, acetone, ethyl acetate and water fractions, showed their pronounced antioxidant properties (in vitro) [115]. The aim of a study conducted by Motallebi Riekandeh et al. [63] was an assessment of the efficiencies of three methods an extraction of antioxidants from *E.caeruleum* inflorescences: the ultrasonically assisted extraction, Soxhlet apparatus and percolation method. Four different in vitro tests were used to evaluate the antioxidant properties of the extracts. *E.caeruleum* inflorescences were gathered in Iran (Sari) in June 2012. Inflorescences were dried and extracted by the three above methods. The antioxidant capacities of extracts are presented in Table 3. Soxhlet extract exhibited a potent effect (IC_50_ = 83.1 μg/mL), then followed an extract obtained by percolation with IC_50_ = 177.3 μg/mL. Each extract demonstrated similar reducing potency (p > 0.05). The activities of these extracts were lower than the reducing power of vitamin C (p < 0.001).

**Table 3:** The antioxidant activities of *E. caeruleum* inflorescence [63]

Extraction method	Fe^2+^ chelating ability, IC_50_ (µg/mL)	NO scavenging activity, IC_50_ (µg/mL)	DPPH radical scavenging, IC_50_ (µg/mL)
Ultrasonic	286 ± 9.2	2416 ± 69.6	188.7 ± 7.2
Percolation	421 ± 13.6	390 ± 11.4	177.3 ± 5.9
Soxhlet	272 ± 6.3	583 ± 9.8	83.1 ± 2.1

^a^IC_50_ of BHA (butylated hydroxyanisole) was 53.8 ± 3.7 μg/mL

^b^IC_50_ for quercetin was 155.0 ± 6.4 μg/mL

^c^EDTA used as control (IC_50_ = 17.4 ± 0.4 μg/mL)

The study of ethyl acetate, methanol, and n-hexane extracts of *E.caeruleum* aerial parts by another group of researchers from Iran showed that their 2,2-diphenyl-1-picrylhydrazyl scavenging activity (%) decreases in order: methanol extract (86.2) > ethyl acetate extract (14.4) > hexane extract (7.1) [16].

### Antibacterial

Antibiotic-resistant bacteria is a global health threat and traditional antibiotics may lose their effectiveness over time due to antibacterial resistance [11, 30, 107, 120]. The development of new natural antimicrobial agents has become a topic of interest in recent years and medicinal plants are a source of antimicrobial compounds with vast therapeutic potential [4, 40, 96]. Secondary plant metabolites, terpenes, flavones, flavonols, some alkaloids and phenylpropanoids, isolated or as a constituent of extracts, have shown promising antimicrobial activity in several studies [4, 41]. The antibacterial activity from leaves and aerial parts of *E. caeruleum* was confirmed by several in vitro investigations. The EO obtained from the leaves of *E. caeruleum* showed high antibacterial activity against six bacterial strains (*Staphylococcus aureus, Bacillus subtilis*, *Streptomyces scabies*, *Erwinia amylovora*, *Xanthomonas axonopodis* pv. citri, and *Klebsiella* sp.) which are essential for plant and human pathogens [18]. In another investigation, Sadiq et al. [80] noted that methanolic extract and its different fractions of *E. caeruleum* aerial parts demonstrated remarkable antibacterial and antifungal activities against six bacterial strains (*Proteus mirabilis, Enterococcus faecalis, Salmonella typhi, Klebsiella pneumonia, Pseudomonas aeruginosa*, and *Escherichia coli*) and three fungal strains (*Aspergillus flavus, Aspergillus fumigatus* and *Aspergillus niger*). The standard drugs used in the antifungal and antibacterial assays were nystatin and ceftriaxone, respectively. All specimens showed pronounced antibacterial activity towards the strains tested. Among the other *E. caeruleum* fractions, the chloroform and ethyl acetate fractions showed superior antibacterial properties. It was observed by the authors that the chloroform fraction of this herb was the most potent one. It exhibited the minimum fungicidal concentration values of 250.64, 350.23, and 233.45 μg/mL against *A. niger*, *A. fumigatus,* and *A. flavus* respectively [80]. Dehghan et al. [17] used an extract of *E. caucasicum* to biosynthesize green nanoparticles of Ag/Fe_3_O_4_.which showed a high inhibition of *Cryptococcus neoformans* at 150 μg/mL. Antimicrobial activities of *E. caeruleum* EO were confirmed against *S. aureus*, *E. coli*, *P. aeruginosa,* and *B. subtilis* using a disc diffusion method by Mohamadipour et al. [61]. In the study of Hamedi et al. [35], the volatile oil of *E. caeruleum* roots showed the highest minimum bactericidal concentration and minimum inhibitory concentration against *B. subtilis* and *S. aureus* (500 μg/mL). According to research findings, EO of *E. caeruleum* contains high levels of trans-pinocarvyl acetate, short-chain fatty acid esters, and certain alcohols such as 1-hexanol and Z-falcarinol, which could play a role in the observed antibacterial activity of them [35].

### Effect on skin permeability

The skin is the largest organ of the body and performs many functions [42, 48, 105]. The pathological conditions determine the change of the skin permeability, respectively it can increase or decrease it, and the effect is more obvious when the skin is damaged, lacking the stratum corneum, has altered keratinization, and the permeability is increased [43, 108]. If the skin is thickened, penetration is low [87]. In the case of dermatoses, characterized by the presence of a defective stratum corneum, there is an increase in absorption through the skin, influencing the local and systemic bioavailability of drug molecules [44, 94]. The investigation of Saeedi and Morteza-Semnani [81] was designed to investigate the ability of different levels of *E. caeruleum* EO to improve the permeation of piroxicam through rat skin and the transdermal absorption of piroxicam. The results showed that no linear correlation was found between the concentration of the EO of *E. caeruleum* and the penetration rate. The increased level of *E. caeruleum* EO (5% w/w) resulted in an increase in the permeability coefficient of piroxicam by nearly 8.56-fold.

### Antihypoxic

*Eryngium caeruleum* inflorescences were collected in the 2012 year and isolated by percolation using methanol [52]. The extract obtained was treated with acetone/methanol/water (3.5/3.5/3) containing 1% formic acid. The aqueous phase was further extracted by ethyl acetate. The organic phase was used as a polyphenols fraction. The methanol extract and polyphenol fraction were used for the study antihypoxic activity (compared to phenytoin). Also, at 400 mg/kg, the polyphenolic fraction increased the death time of experimental animals. It had a comparative advantage over phenytoin (55 min, p > 0.05). The polyphenolic fraction was found to be more potent compared to the methanolic extract [52].

### Anti-inflammatory

**Fig. 4:**
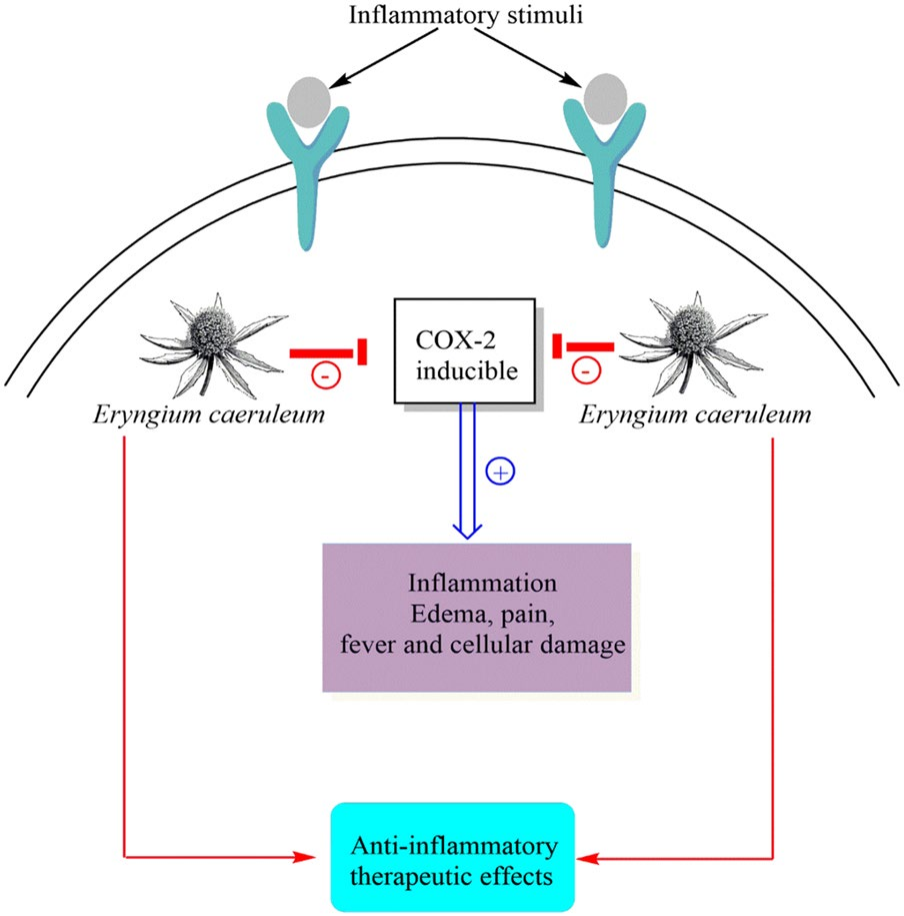
Anti-inflammatory effects of *E. caeruleum*

The study by Habibi et al. [34] showed that a dose of 200 and 400 mg/kg of ethyl acetate extract of *E. caeruleum* was effective in inducing an anti-inflammatory effect while reporting that a higher dose, causes a decrease in this effect. Furthermore, *E. caeruleum* exhibited a comparable anti-inflammatory effect to celecoxib as a cyclooxygenase (COX)-2 inhibitor (Fig. 4), however, its undesirable reaction is less than that of the chemical drugs, therefore, the Habibi et al. [34] believe that suggested some inflammatory diseases such as rheumatoid arthritis. It can be hypothesized that the anti-inflammatory properties of *E. caeruleum* might be linked to a high content of flavonoids and phenol like quercetin.

### Anti-diabetes

Diabetes is a chronic non-communicable disease that occurs when the pancreas can no longer produce insulin, or when the body can no longer use insulin [76, 84]. The increasing frequency of hyperglycemia of various etiologies and the side effects of many synthetic hypoglycemic drugs have led to a shift toward phytotherapy [75, 91].

**Fig. 5:**
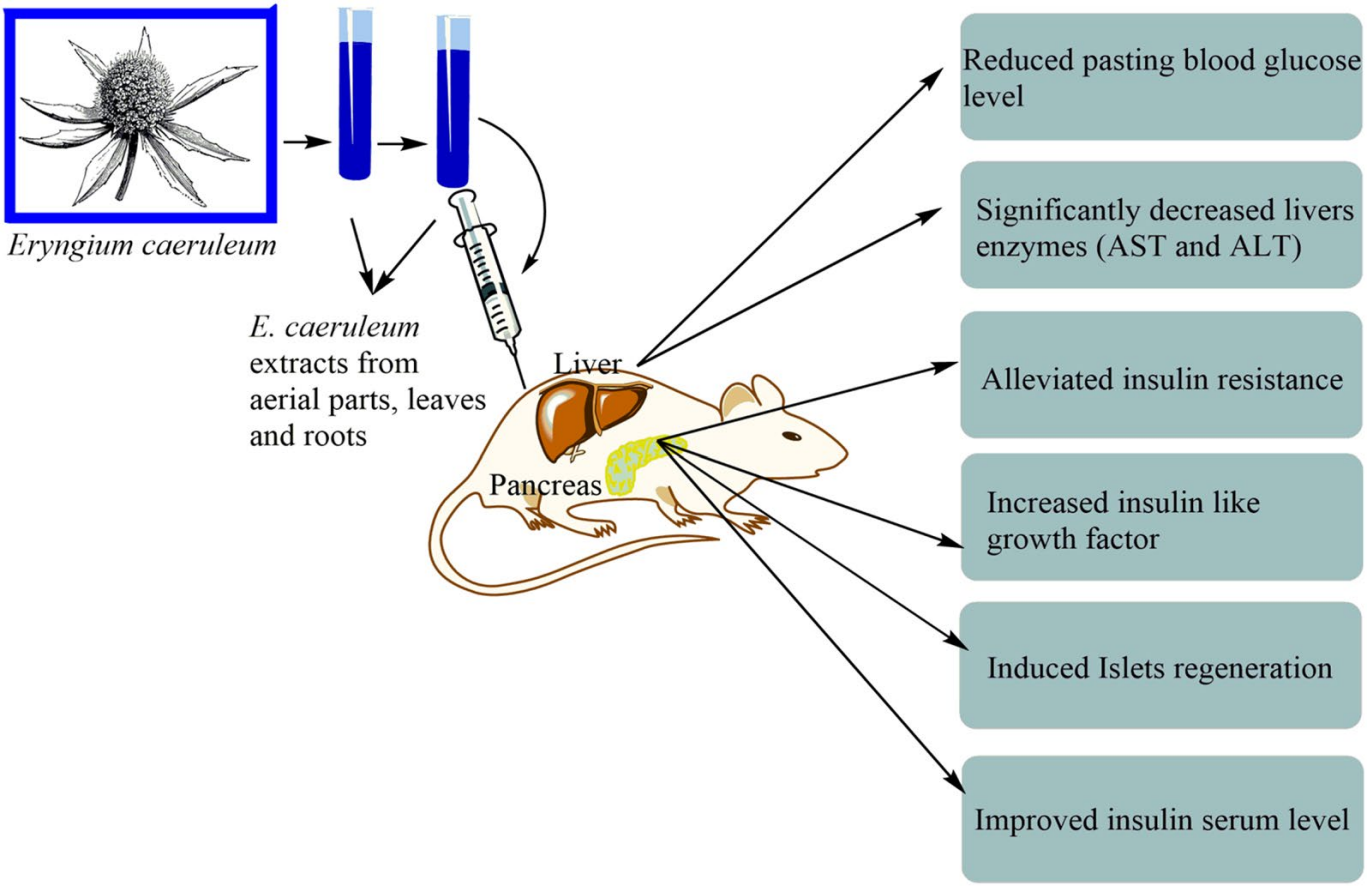
Antidiabetic effects of *E. caeruleum*

Dehghan et al. [16] have reported that ethyl acetate, methanolic, and n-hexane extracts of aerial parts of *E. caeruleum* possess an anti-diabetic effect. The methanolic extract showed a higher effect. Rehman et al. [79] reported two new flavone glycosides derived from *E. caeruleum* aerial parts. These compounds were analyzed in vitro against aldose reductase (ALR) of two types (ALR1 and ALR2). Also, flavonoid 2 showed a very significant inhibitory activity against both enzymes, while the effect on ALR1 was the highest [79]. Afshari et al. [2] studied the anti-diabetetes effect of *E. caeruleum* leaves hydro-alcoholic (70:30) extract in a model of type 2 diabetes mellitus induced by nicotinamide-streptozotocin in Wistar rats. Insulin-related indices, insulin secretion, and lipid profile were affected by the administration of *E. caeruleum* extract. It also revealed a significant decrease in liver enzymes. Additionally, *E. caeruleum* enhanced levels of serum insulin-like growth factor in a dose-dependent manner. Overall, it appears that *E. caeruleum* causes diabetes in rats in a dose-dependent manner, with increasing doses exhibiting greater benefits. In a study performed in a streptozotocin-induced diabetes rat model, the hydro-alcoholic (70:30) extract of aerial branches and roots of *E. caeruleum* improved glycemic and lipid profiles [86]. Pancreatic histology results revealed that extracts from the root and aerial branch (800 mg/kg) induced islets regeneration. In another investigation, the administration of metformin and *E. caeruleum* extract alone or in combination ameliorate types of morphologic injury to renal tubular cells in diabetic rats [31] (Fig. 5).

### Hepatoprotective and renoprotective

The liver is an organ with a series of very important roles in the body: purification, detoxification, bile secretion necessary for intestinal digestion, having a decisive role in the synthesis processes, degradation and storage of organic and inorganic substances absorbed at the level of intestinal [20, 49, 116]. Some liver conditions can be treated with natural remedies and others with medication [14, 20, 51, 68]. Fattahi and Hemayatkhah Jahromi [28] investigated the protective role of *E. caeruleum* extract on hepatotoxicity in a mice model using tricyclazole (TCZ). The extract treatment reduced bilirubin levels and liver enzymes (aminotransferase and phosphatase alkaline) and increased albumin levels (p < 0.05) in TCZ-treated mice. Methanol/water (80/20 w/w) extract of *E. caeruleum* aerial parts (200 and 400 mg/kg/day). Intraperitoneally administered for ten consecutive days, provoked a renoprotective action [27].

### Anti-convulsant

Epilepsy is a syndrome of cerebral distress, which is manifested by epileptic seizures [10, 98, 109]. There are two factors in its genesis: convulsive predisposition and the existence of an anatomical or biochemical brain injury [39, 47]. The medical term for seizures refers to abnormal, involuntary contractions of the muscles as a result of changes in the electrical activity of the brain and which generally occur in certain epileptic diseases [9, 98]. The objective of the study performed by Ebrahimzadeh et al. [23] was to assess the anticonvulsant effects of polyphenolic and methanolic extracts (250, 500 and 1000 mg/kg) of *E. caeruleum* inflorescences on pentylenetetrazole-induced seizures and maximal electroshock in mice. The effect of the polyphenolic extract was more potent. According to the authors, a mechanism of GABAergic potentiation or free radical scavenging might be possible for these pharmacological abilities [23].

## Applications in the food industry

Raeisi et al. [78] demonstrated that *E. caeruleum* leaves extract (80% ethanol) had good potential for use as a natural preservative for shelf-life extension of fishery products. They examined the effects of *E. caeruleum* extract (after removing alcohol) on rancidity indices and lipid oxidation, sensory and microbiological qualities during refrigerated storage (4 °C) of silver carp (*Hypophthalmichthys molitrix*). The inclusion of plant extracts considerably retarded oxidative damage of silver carp fillets during the period of storage. Further, the findings indicated that the natural constituents from *E. caeruleum* extract were effective in decreasing bacterial growth within the fish fillets during the period of refrigerated storage. In addition, it was observed that 4% *E. caeruleum* had a beneficial effect on the sensory characteristics of the fillets [78]. Golmohammadi and Khademi shurmasti [32] investigated the effect of *E. caeruleum* extract on chicken fillets shelf life. The examination was carried out with uncoated chicken fillets (control), and fillets that were coated with xanthan and guar gums alone or in combination with *E. caeruleum* extract in during 12-day refrigerated storage. The findings revealed that *E. caeruleum* extract markedly (p < 0.05) enhanced the antibacterial function of guar coating. Also, the effect of *E. caeruleum* extract on improving the efficiency of edible guar coating resulted in a significant decrease in total volatile nitrogen compounds of the fillet (p < 0.05) [32].

## Clinical studies

A blinded, randomized, placebo-controlled clinical trial involving 169 women between the ages of 15 and 30 years was aimed at studying the safety and effectiveness of syrup of *E. caeruleum* in the treatment of primary dysmenorrheal (Iranian Registry of Clinical Trials ID: IRCT2015082823789N1) [7]. The dosage was determined by the investigators to consist of 5 ml of syrup administered three times a day (15 ml/day), starting one day prior to the onset of bleeding and continuing for five days over two menstrual cycles. There have been no reported serious adverse reactions. *E. caeruleum* syrup provided relief from dysmenorrhea as efficiently as Ibuprofen.

## Limitations

A limiting therapeutic aspect is the lack of studies on the *E. caeruleum* toxicity, the side effects that may occur during prolonged administration and the potential interactions with the medication administered concomitantly. In addition, the concentration of active ingredients varies depending on the cultivation area and soil characteristics. Also, the reduced bioavailability of the active ingredients is an important factor in the therapeutic efficacy. Therefore, the inclusion of phytocompounds in some pharmaceutical nanoformulations can increase their bioavailability. Saleh et al. [82] synthesized bio-compatible palladium nanoparticles via *E. caeruleum* leaves extract. These showed antibacterial activity against multidrug-resistant bacteria, high antioxidant activity and not showed hemolytic activity [82].

## Conclusions and prospects

This article reports, explores and highlights published evidence on *E. caeruleum* regarding its botanical characteristics and traditional uses, its phytochemical composition, its cultivation, and its bioactivity and pharmacological properties as a promising medicinal plant. *E. caeruleum* leaves, traditionally are used to flavor cooked vegetables in various local products. In traditional medicine, the aerial parts of *E. caeruleum* are largely used for treating pulmonary disease, asthma, epigastric, and hypochondriac pain. The roots of the plant cause a diuretic effect, effective against kidney and bladder stones, as an analgesic for rheumatoid arthritis. Studies show that *E. caeruleum* is a potential preventative and medicinal plant. Extracts from aerial part, leaves and roots containing EO, phenolic compounds, saponins, protein, amino acids, fiber, carbohydrates, and mineral elements have antioxidant, antimicrobial, antidiabetic, antihypoxic, anti-inflammatory, and other properties. These effects show that *E. caeruleum* could be considere a novel herbal-derived medicine for the management of certain diseases. Nevertheless, stringent investigation studies are necessary to determine its effectiveness by examining its toxicological, pharmacological, chemical, and therapeutic effects. Future studies must evaluate new methods of obtaining and administering some drugs/nutritional supplements to increase the absorption and bioavailability of the bioactive compounds from the E. caeruleum species. Thus, new pharmaceutical forms such as nanoparticles should be evaluated in more detail in animals through preclinical in vivo and in vitro experimental studies. Translational medicine studies and clinical studies are also needed to accurately establish the effective dose and the method of administration in humans.

## Data Availability

Yes.
